# Effects of dietary lysine levels on plasma free amino acid profile in late-stage finishing pigs

**DOI:** 10.1186/s40064-016-2463-3

**Published:** 2016-06-24

**Authors:** Naresh Regmi, Taiji Wang, Mark A. Crenshaw, Brian J. Rude, Guoyao Wu, Shengfa F. Liao

**Affiliations:** Department of Animal and Dairy Sciences, Mississippi State University, Mississippi State, MS 39762 USA; Department of Animal Science, Texas A&M University, College Station, TX 77843 USA

**Keywords:** Lysine, Dietary lysine supply, Amino acid profile, Plasma, Swine, Arginine

## Abstract

Muscle growth requires a constant supply of amino acids (AAs) from the blood. Therefore, plasma AA profile is a critical factor for maximizing the growth performance of animals, including pigs. This research was conducted to study how dietary lysine intake affects plasma AA profile in pigs at the late production stage. Eighteen crossbred (Large White × Landrace) finishing pigs (nine barrows and nine gilts; initial BW 92.3 ± 6.9 kg) were individually penned in an environment controlled barn. Pigs were assigned randomly to one of the three dietary treatments according to a randomized complete block design with sex as block and pig as experiment unit (6 pigs/treatment). Three corn- and soybean meal-based diets contained 0.43 % (lysine-deficient, Diet I), 0.71 % (lysine-adequate, Diet II), and 0.98 % (lysine-excess, Diet III) l-lysine, respectively. After a 4-week period of feeding, jugular vein blood samples were collected from the pigs and plasma was obtained for AA analysis using established HPLC methods. The change of plasma lysine concentration followed the same pattern as that of dietary lysine supply. The plasma concentrations of threonine, histidine, phenylalanine, isoleucine, valine, arginine, and citrulline of pigs fed Diet II or III were lower (*P* < 0.05) than that of pigs fed Diet I. The plasma concentrations of alanine, glutamate, and glycine of pigs fed Diet II or III were higher (*P* < 0.05) than that of pigs fed Diet I. The change of plasma leucine and asparagine concentrations followed the patterns similar to that of plasma lysine. Among those affected AAs, arginine was decreased (*P* < 0.05) in the greatest proportion with the lysine-excess diet. We suggest that the skeletal muscle growth of finishing pigs may be further increased with a lysine-excess diet if the plasma concentration of arginine can be increased through dietary supplementation or other practical nutritional management strategies.

## Background

The primary goal of animal agriculture (e.g., the swine industry) is to grow skeletal muscle (the major component of the body) and, thus, to produce meat with high-quality protein for human consumption (Wu et al. 2014a, b). Previous research has shown that growth and development of muscle essentially requires dietary supply of all proteinogenic amino acids (AAs) (Wu et al. 2014c). There are 20 different proteinogenic AAs in the diets that are commonly fed to pigs. The main function of these AAs is to serve as building blocks for biosynthesis of animal body proteins, such as muscle proteins that are the largest AA reservoir in the body (Hou et al. 2015). Like many other body proteins, muscle proteins undergo continuous turnover; namely old, damaged, or unneeded proteins are degraded and new proteins are de novo synthesized (Liao et al. 2015). Therefore, a constant supply of sufficient AAs to living cells from the blood is required to ensure protein accretion in skeletal muscle of growing pigs, i.e., the rate of protein biosynthesis is greater than the rate of protein degradation.

Protein is a polymer of AA residues joined together by peptide bonds. Each protein has a unique linear AA sequence and, thus, fixed ratios of different AAs (Wu 2013). For maximal protein biosynthesis in skeletal muscle, various free AAs must be available simultaneously in certain ratios that match muscle protein AA composition (Christensen 1964; Liao et al. 2015). Unbalanced ratios of AAs can diminish muscle protein biosynthesis, which means if one proteinogenic AA is deficient, protein synthesis will be reduced. This will result in the net degradation of muscle proteins to release AAs for use by other important organs, such as the brain, liver, small intestine, and lymphoid organs (Waterlow 1969; Gustafson et al. 1986; Wu 2014).

From nutrient metabolism standpoint, AAs are directly and indirectly related to each other within the overall metabolic pathways, and plasma free AA profile reflects the sum of metabolic flow of nutrients and their metabolites from all tissues and organs (Christensen 1964; Liao et al. 2015). In many physiological circumstances, tissue uptake of free AAs and further the metabolism of AAs largely depend on the concentrations of plasma free AAs (Cynober 2002). Thus, in pigs, plasma free AA profile, which can be readily monitored, may provide animal nutritionists with indication of AA metabolic status, a key index of body protein turnover. Because plasma free AAs are principally the direct source of AA supply for muscle protein biosynthesis and plasma AA profile does not parallel dietary AA profile (Wu 2014), the knowledge of plasma AA profile may be more imperative than the knowledge of dietary AA profile to evaluate the adequacy of AAs for tissue protein biosynthesis.

Dietary AA deficiency is usually overcome either by increasing dietary crude protein intake or by adding crystalline AAs. Lysine is the first limiting AA in typical grain-based swine diets (Liao et al. 2015). It has been reported that a deficiency of dietary lysine can reduce the expression of AA transporters in the small intestine (He et al. 2013) and alter the plasma concentrations of AAs (Morrison et al. 1961; Yun et al. 1991; Zeng et al. 2013), leading to a series of interconnected consequences for swine health and productivity. Thus, there is a pressing need to further study the effect of dietary lysine on plasma AA profile in order to explore the metabolic interactions between lysine and other AAs and to identify an optimal plasma AA profile to maximize pig growth performance via the best combination and supply of dietary AAs. Therefore, this study was conducted to investigate the effect of three critical levels of dietary lysine on plasma concentrations of free AAs in late-stage finishing pigs.

## Methods

### Animal trial procedures

Eighteen crossbred (Large White × Landrace) nursery pigs (nine barrows and nine gilts), with average body weight (BW) 20.5 ± 0.9 kg, were purchased from a local commercial swine farm and transferred to an environment controlled swine barn at the Leveck Animal Research Center of Mississippi State University. After arrival, pigs were phase-fed commercial nursery and grow-finish diets until their BW reached 92.3 ± 6.9 kg, during which period, pigs were allowed ad libitum access to feed and water. Pigs were then randomly assigned into 18 individual feeding pens, and further randomly assigned to three dietary treatment groups according to a randomized complete block experimental design with gender as block and pig as experimental unit (three barrows and three gilts per treatment).

A corn- and soybean meal-based diet (Diet I; a lysine-deficient diet) was formulated to meet or exceed the NRC (2012) recommended requirements (on a per-kg-diet basis for 100–135 kg BW pigs) of various nutrients including crude protein but not lysine. Diet II (a lysine-adequate diet) and Diet III (a lysine-excess diet) were formulated by adding l-lysine monohydrochloride (98.5 %; Archer Daniels Midland Co., Quincy, IL) to Diet I at the expense of corn at ratios of 0.35 and 0.70 %, respectively (Table 1). Calculated total lysine contents (as-fed basis) in Diets I, II, and III were 0.43, 0.71, and 0.98 %, respectively. No effort was made to maintain a constant amino acid balance. To confirm the contents of major nutrients, samples of the three diets were submitted to the Essig Animal Nutrition Laboratory at Mississippi State University for proximate analysis, and to Guoyao Wu’s laboratory at Texas A&M University for AA analysis (Dai et al. 2014). Shown in Table 2 are analyzed compositions of various nutrients in the three experimental diets.

**Table 1:** Composition of three corn- and soybean meal-based diets for the finishing pigs

Item	Experimental diet		
	Diet I	Diet II	Diet III
*Ingredient (%)*			
Corn	90.844	90.494	90.144
Soybean meal	6.400	6.400	6.400
Canola oil	0.800	0.800	0.800
l-Lysine-HCl^a^	0.000	0.350	0.700
dl-Methionine^b^	0.040	0.040	0.040
l-Threonine^a^	0.090	0.090	0.090
l-Tryptophan^c^	0.035	0.035	0.035
Limestone	0.650	0.650	0.650
Dicalcium phosphate	0.900	0.900	0.900
Salt	0.200	0.200	0.200
Mineral premix^d^	0.033	0.033	0.033
Vitamin premix^e^	0.008	0.008	0.008
*Calculated energy and nutrient* ^f^			
Dry matter (%)	90.0	90.0	90.0
Metabolizable energy (kcal/kg)	3319	3323	3326
Net energy (kcal/kg)	2371	2375	2378
Crude protein (%)	10.45	10.75	11.0
SID lysine (%)	0.32	0.60	0.87
SID methionine + cysteine (%)	0.47	0.47	0.47
Total Ca (%)	0.46	0.46	0.46
Total P (%)	0.43	0.43	0.43
Available P (%)	0.26	0.26	0.26

As fed basis

*SID* standardized ileal digestible

^a^ l-Lysine-HCl (98.5 %) and l-threonine (98.5 %) were donated by Archer Daniels Midland Co., Quincy, IL)

^b^DL-Methionine (99.0 %; Rhodimet, NP 99) was donated by Adisseo USA Inc. (Alpharetta, GA)

^c^ l-Tryptophan (99.0 %) was donated by Ajinomoto Heartland, Inc. (Chicago, IL)

^d^Swine Trace Mineral Mix (No. 85) was donated by Prestage Farms of Mississippi, Inc. (West Point, MS) that contained 13.2 % Ca, 1.0 % Cu, 8.0 % Fe, 5.0 % Mn, 10.0 % Zn, 500 ppm I, and 300 ppm Se

^e^Vitamin Premix for Market Swine was donated by Prestage Farms of Mississippi, Inc. (West Point, MS) that contained per kilogram 22.05 million IU vitamin A, 3.31 million IU vitamin D_3_, 66,138 IU vitamin E, 88 mg vitamin B_12_, 220 mg biotin, 8818 mg menadione, 15,432 mg riboflavin, 61,728 mg d-pantothenic acid, and 88,183 mg niacin

^f^Only the selected major nutrients are listed

**Table 2:** Analyzed nutrient composition of the three corn- and soybean meal-based diets

Nutrient and energy^a^	Experimental diet		
	Diet I	Diet II	Diet III
Dry matter (%)	87.10	87.10	87.10
Gross energy (kcal/kg)	3663	3608	3559
Crude protein (%)	9.77	10.60	10.86
*Individual amino acids (%)*			
Lysine	0.42	0.70	1.01
Aspartate	0.98	0.97	0.98
Asparagine	0.74	0.75	0.75
Glutamate	1.01	1.03	1.03
Glutamine	1.42	1.44	1.43
Serine	0.53	0.52	0.54
Histidine	0.34	0.33	0.34
Glycine	0.61	0.62	0.62
Threonine	0.5	0.51	0.5
Arginine	0.73	0.74	0.74
Alanine	0.81	0.83	0.82
Tyrosine	0.52	0.52	0.53
Tryptophan	0.14	0.13	0.14
Methionine	0.25	0.25	0.26
Valine	0.65	0.66	0.65
Phenylalanine	0.66	0.65	0.66
Isoleucine	0.52	0.52	0.53
Leucine	1.42	1.44	1.45
Cysteine	0.26	0.26	0.3
Proline	1.08	1.07	1.09

^a^Amino acids were analyzed as described by Dai et al. (2014) in Guoyao Wu’s laboratory, Texas A&M University (College Station, TX), while energy, crude protein and dry matter were analyzed at the Essig Animal Nutrition Laboratory, Mississippi State University (Starkville, MS). All values are expressed on the as-fed basis

Pigs were allowed ad libitum access to the experimental diets and fresh water throughout the trial which lasted for a total of 4 weeks. All pigs, feeders, and waterers were checked 2–3 times daily. The BW of pigs were measured at the beginning and the end of the 4 week period for calculation of average daily gain (ADG). All experimental protocols involving caring, handling, and treatment of pigs were approved by Mississippi State University Institutional Animal Care and Use Committee.

### Sample collection and laboratory analyses

At the conclusion of the 4 week feeding trial, blood samples (approximately 10 mL/pig) were collected with vacutainer tubes containing anticoagulant (i.e., EDTA) by venipuncture of jugular veins of pigs between 06:00 and 08:00 am. Immediately before starting blood collection, all the leftover feeds were removed from all the feeders so that no pigs had access to the feed during the blood collection time. Thus, venous blood samples were obtained in a non-fasting state. Immediately after collection, blood samples were placed onto ice until plasma was separated by centrifugation for 16 min at 800 × *g* and 4 °C. Plasma samples were stored in 200-μL aliquots at −80 °C until the laboratory analyses of AAs were conducted.

Concentrations of plasma free AAs were determined using high-performance liquid chromatography (HPLC) methods (Wu 1993; Liao et al. 2005; Dai et al. 2014). Briefly, after a pre-column derivatization of plasma AAs with *o*-phthaldialdehyde, the samples were separated on a Supelco 3-μm reversed-phase C18 column (4.6 × 150 mm, i.d.) guarded by a Supelco 40-μm reversed-phase C18 column (4.6 × 50 mm, i.d.). The HPLC mobile phase consisted of solvent A (0.1 M sodium acetate/0.5 % tetrahydrofuran/9 % methanol; pH 7.2) and solvent B (methanol), with a combined total flow rate of 1.1 mL/min. A gradient program with a total running time of 49 min was developed for satisfactory separation of AAs. Proline and cysteine were analyzed using two other different methods (Wu 1997; Wu and Meininger 2008).

### Statistical analysis

Data were analyzed using the General Linear Model (GLM) procedure of SAS (version 9.3; SAS Institute Inc. Cary, NC) for two-way ANOVA with gender (block) and dietary lysine level as two main effects and individual pigs as experiment units. Means were separated with PDIFF (adjust = T) option as preplanned. Probability values (*P*) less than 0.05 were considered as significant differences and *P* values between 0.05 and 0.10 were considered as tendencies to be different. Because there was no effects of block and the block × lysine level interaction detected, only the main effect of lysine level was presented for results.

## Results

As shown in Table 3, there were no differences in the initial BW among the three treatment groups fed three different diets. At the end of the trial, the final BW of pigs fed Diet II or III were greater (*P* < 0.05) than that of pigs fed Diet I, and there was no difference in the final BW between pigs fed Diets II and III. The ADG changed in the same manner as that of final BW changed among the treatment groups. The ADG of pigs fed Diet II or III was greater (*P* < 0.05) than that of pigs fed Diet I, although there was no difference in the ADG between pigs fed Diets II and III.

**Table 3:** Effects of dietary lysine levels on body weight gain of the late-stage finishing pigs

Parameters^1^	Treatment group			SE^2^	*P* value^3^
	Diet I	Diet II	Diet III		
Initial BW (kg)	93.0	91.8	92.5	3.07	0.962
Final BW (kg)	120.8^a^	130.5^b^	130.6^b^	4.13	0.189
ADG^4^ (kg/day)	0.99^a^	1.38^b^	1.36^b^	0.06	0.004

^1^The total calculated lysine contents in Diets I, II, and III were 0.43, 0.71, and 0.98 % (as-fed basis), respectively

^2^SE = pooled standard error of mean (n = 6)

^3^ *P* value was obtained from the ANOVA F test

^4^ADG, average daily gain

^a,b^Means within a row that do not share a same superscript differ (*P* < 0.05 or 0.01)

Plasma free AA profiles of pigs fed Diets I, II, and III are shown in Table 4. From this table, it can be seen that the concentration of glycine was the highest (917–1324 nmol/mL), whereas the concentration of aspartate was the lowest (17.0–18.3 nmol/mL) in the plasma of the pigs. While the total AA (TAA) concentration ranged from 4153 to 4668 nmol/mL, the concentration of total non-lysine essential AAs (EAA, excluding lysine) ranged from 841 to 1132 nmol/mL, and the total “nonessential AA” (NEAA) concentration ranged from 2930 to 3444 nmol/mL. While the plasma concentrations of 11 AAs, aspartate, β-alanine, cysteine, glutamine, methionine, ornithine, proline, serine, taurine, tryptophan, and tyrosine, were not affected, the plasma concentrations of 13 AAs, lysine, leucine, arginine, citrulline, histidine, threonine, isoleucine, valine, phenylalanine, alanine, glutamate, glycine and asparagine, were affected (*P* < 0.05) by dietary treatment, and so were concentrations of total EAA, total NEAA, and TAA (Table 4).

**Table 4:** The concentrations of free amino acids in the plasma of finishing pigs fed three levels of dietary lysine^1^

Amino acids (nmol/mL)^2^	Treatment group			SE^3^	*P* value^4^
	Diet I	Diet II	Diet III		
*Lysine*	91.0^a^	317.6^b^	501.4^c^	42.5	<0.0001
*Total EAA*	1132.4^b^	861.6^a^	840.7^a^	44.5	0.0009
Leucine	179.4^a^	195.9^a,b^	200.1^b^	6.32	0.081
Histidine	124.1^b^	89.4^a^	88.3^a^	8.21	0.011
Phenylalanine	106.4^b^	56.6^a^	58.2^a^	11.3	0.010
Isoleucine	108.4^b^	77.1^a^	77.9^a^	5.32	0.001
Threonine	258.4^b^	149.7^a^	138.8^a^	11.2	<0.0001
Valine	209.1^b^	142.8^a^	144.6^a^	10.1	0.0004
Methionine	64.4	59.7	58.3	3.03	0.352
Tryptophan	82.1	90.2	74.6	6.12	0.231
*Total NEAA*	2929.5^a^	3444.0^b^	3325.5^b^	107.3	0.013
Arginine	228.1^b^	185.5^a^	159.6^a^	13.3	0.008
Citrulline	102.2^b^	76.3^a^	63.1^a^	6.69	0.003
Alanine	400.9^a^	509.1^b^	509.8^b^	31.9	0.045
Glutamate	89.5^a^	128.6^b^	106.0^b^	12.3	0.113
Glycine	917.4^a^	1324.0^b^	1308.4^b^	52.6	<0.0001
Asparagine	69.4^a^	78.3^a^	103.9^b^	7.98	0.021
Aspartate	17.0	18.1	18.3	1.87	0.869
β-Alanine	47.7	51.6	44.4	3.71	0.413
Cysteine, total^5^	193.8	203.3	199.8	4.72	0.385
Glutamine	459.4	466.5	420.8	17.3	0.166
Ornithine	110.2	93.8	108.0	8.00	0.319
Proline	286.2	290.2	284.0	5.57	0.734
Serine	190.5	235.1	233.0	24.0	0.358
Taurine	189.1	174.8	147.8	15.2	0.185
Tyrosine	108.2	102.5	102.5	4.57	0.611
*Total AA*	4152.9^a^	4623.2^b^	4667.6^b^	146.2	0.053

^1^Calculated total lysine contents (as-fed basis) in Diets I, II, and III were 0.43, 0.71, and 0.98 %, respectively

^2^EAA = essential amino acids excluding lysine, NEAA = “non-essential amino acids”, and total AA include lysine, total EAA, and total NEAA

^3^SE = pooled standard error of the mean, n = 6

^4^ *P* values were obtained from the ANOVA F test

^5^Total cysteine (cysteine + ½ cystine)

^a,b,c^Means within a row that do not share a same superscript differ (*P* < 0.05)

As expected, the change of plasma lysine concentration followed the same pattern as that of dietary lysine supply. The plasma concentration of lysine in pigs fed Diet II was greater (*P* < 0.05) than that in pigs fed Diet I, and the plasma lysine concentration in pigs fed Diet III was greater (*P* < 0.05) than that in pigs fed Diet II (Table 4). The plasma concentration of leucine was not different between pigs fed Diets I and II or between pigs fed Diets II and III. However, pigs fed Diet III had a greater (*P* < 0.05) plasma concentration of leucine, compared with pigs fed Diet I.

The change of plasma concentrations of 5 EAA (histidine, threonine, phenylalanine, isoleucine, and valine) as well as total EAA followed a different pattern than that of lysine or leucine. The plasma concentrations of these EAA in pigs fed Diet II or III were lower (*P* < 0.05) than those in pigs fed Diet I, and there were no differences between pigs fed Diets II and III. On the contrary, the plasma concentrations of 3 NEAA (alanine, glutamate, and glycine), as well as total NEAA, in pigs fed Diet II or III were greater (*P* < 0.05) when compared to pigs fed Diet I, with no difference being detected between pigs fed Diets II and III. However, the change of plasma concentrations of arginine and citrulline followed the same pattern as that of total EAA, but not that of total NEAA. There was no difference (*P* = 0.44) between pigs fed Diets I and II in the plasma concentration of asparagine, and the plasma concentration of asparagine in pigs fed Diet III was greater (*P* < 0.05) than that in pigs fed Diet I or II.

Although the plasma concentrations of 2 EAA (methionine and tryptophan) and 9 NEAA [aspartate, β-alanine, total cysteine (cysteine + ½ cystine), glutamine, ornithine, proline, serine, tyrosine and taurine] did not differ among the three groups of pigs (Table 4), the alteration in the plasma concentration of TAA followed the same pattern as that of total NEAA.

## Discussion

Although dietary supply of proteins and AAs are the ultimate source of free AAs in the plasma of pigs, the free AAs are directly and indirectly related to each other within the overall nutrient metabolic pathways (Wu 2013). Importantly, the plasma free AA profile reflects the dynamic state of the metabolic flux of AAs absorbed from the small intestine, as well as the rates of their utilization and intracellular protein turnover in the whole body (Bongiovanni and Feinerman 2003; Shikata et al. 2007; Liao et al. 2015). Theoretically, dietary supply of lysine at different levels will affect not only the plasma concentration of lysine, but also the plasma concentrations of other AAs. However, the patterns of change in plasma concentrations of all free AAs have not been thoroughly studied in pigs. Results of the present study indicate five distinct patterns of change in the plasma concentrations of free AAs in the late-stage finishing pigs fed ad libitum during the experimental period.

As pattern 1, the plasma concentration of lysine increased as dietary lysine increased from a deficient to an adequate level, as well as from an adequate to an excess level. This pattern of change paralleled with the pattern of dietary lysine supply, indicating that the capacity of small-intestinal cationic AA transporters is not a limiting factor for absorption of dietary lysine within the range of dietary concentration of up to approximately 1.0 %. This result is similar to the findings of Braude et al. (1974), Roy et al. (2000), and Zeng et al. (2013) in growing pigs and Chen et al. (1978) in lactating sows. Lysine is known to be very conservative in terms of catabolism (Liao et al. 2015) and its oxidation rate is relatively slower compared to the oxidation rates of other EAA (Yang et al. 1968; Yamashita and Ashida 1969; Chu and Hegsted 1976; Blemings et al. 1989; Wu 2014). These characteristics of lysine metabolism may explain why the plasma concentration of lysine increased linearly with the increased concentrations of dietary lysine.

The second pattern of change was followed by 5 EAA, histidine, threonine, phenylalanine, isoleucine, and valine, as well as total EAA (Table 4), which is in agreement with the findings of Zimmerman and Scott (1965) for chicks, who reported that dietary lysine deficiency resulted in increased plasma concentrations of several AAs, such as histidine, threonine, phenylalanine, isoleucine, leucine, and tyrosine in the birds. Roy et al. (2000) found that plasma concentrations of threonine, isoleucine, valine, and taurine were greater in growing barrows fed lysine deficient diets. This pattern of change firmly supports the limiting AA concept in swine nutrition, which states that if one EAA is deficient in a diet, body protein synthesis will not continue beyond the level of this EAA, which is lysine in the case of this study. When protein synthesis in the whole body (particularly, the skeletal muscle) is limited, the concentrations of EAA in the plasma of pigs would increase. When dietary level of lysine increased from deficient (as in Diet I) to adequate (as in Diet II) level, protein synthesis also increased until reaching the level of next limiting EAA, and this is why plasma concentrations of the aforementioned 5 EAA as well as total EAA were decreased in Diet II pigs, when compared with Diet I pigs. When the dietary level of lysine continued to increase from the adequate (as in Diet II) to the excess (as in Diet III) level, the plasma concentrations of these 5 EAA did not continue to decline, indicating that protein synthesis could not continue to increase in lysine-excess pigs possibly due to inadequate provision of a second limiting AA.

The change pattern of plasma arginine and citrulline concentrations also followed the second pattern of change as that of the aforementioned 5 EAA, which was reflected in the ADG change of these pigs. The ADG of pigs increased as dietary lysine increased from deficient to adequate level, but did not further increase as dietary lysine increased from the adequate to the excess level (Table 3). In theory, the ADG associated with lysine-excess diet could be further increased if greater concentrations of the 5 EAA and arginine in the plasma could be achieved. Among these AAs, it is currently not clear which one was the most limiting. Of note, arginine, a conditionally essential AA for pigs (Hou et al. 2015), was decreased in the greatest proportion and might be the first limiting AA for the pigs in the lysine-excess group.

In mammals, although arginine can be formed from citrulline by a variety of extra-hepatic cells via the arginine-citrulline cycle (Wu and Flynn 1993; Wu and Meininger 1993), citrulline and arginine in adult pigs are mainly synthesized from glutamine, glutamate and proline in enterocytes (Wu et al. 1994; Wu and Knabe 1995; Wu 1997). The citrulline released from the enterocytes can be taken up primarily by kidneys from blood for synthesis of arginine in post-weaning mammals, including pigs (Wu and Morris 1998). In mammals, arginine is catabolized via multiple pathways, with the production of urea and creatine being its major products (Hou et al. 2016a; Wu et al. 2016).

The negative relationship between lysine and arginine, however, has been studied in several animal species, and there are three contributing factors that may be responsible for this lysine-arginine antagonism: (1) the competitive inhibition of arginine absorption from intestinal lumen (Harper et al. 1970; Kamin and Handler 1952; Larsen et al. 1964), reabsorption from kidney tubules, and transport of basic AAs by other tissues (Jones et al. 1967; Nesheim 1968; Boorman 1971; Popolo et al. 2014; Monné et al. 2015); (2) the induction of kidney arginase (Austic and Nesheim 1970; Robbins and Baker 1981); (3) the inhibition of liver transaminase (Austic and Nesheim 1971); and (4) the changes in the synthesis of arginine metabolites such as nitric oxide, homoarginine, creatine, and polyamines (Hu et al. 2015; Kayacelebi et al. 2015; Yang et al. 2015), which are of enormous importance in cell nutrition and metabolism (Agostinelli 2014; Bernstein et al. 2015; Tan et al. 2015; Tsikas and Wu 2015; Wu et al. 2015).

In profiling AAs of muscle protein in finishing pigs, Cai et al. (2010) reported that arginine constitutes about 4.88 % of total protein of *longissimus dorsi* muscle. However, in our present study, we found that arginine represented 5.5, 4.01 and 3.42 % of total plasma free AAs in lysine-deficient, -adequate, and -excess diets, respectively. These ratios indicated that, in the lysine-excess group, the supply of arginine from the plasma to skeletal muscle might be a limiting factor for protein synthesis. Thus, increasing the plasma concentration of arginine, possibly through dietary arginine supply, may increase the response of pig muscle growth to dietary lysine supplementation. This strategy is promising, because dietary arginine enhances lean tissue growth and reduces whole-body white fat in growing-finishing pigs (Tan et al. 2009) and supplementing up to 2 % arginine to a typical corn- and soybean meal-based diet is safe for growing pigs (Hu et al. 2015; Wu et al. 2016).

The third pattern of change in plasma AA concentrations was followed by 3 NEAA (alanine, glutamate, and glycine) and total NEAA (Table 4). A possible reason for the decreased plasma concentrations of these 3 NEAA in the lysine-deficient group might be because of the reduced rates of their synthesis and/or the increased rates of their oxidation in a tissue-specific manner. In lysine-deficient pigs, the rate of muscle protein synthesis is limited so that there may be no need to have more NEAA as building blocks for protein synthesis. Because the de novo production of NEAA requires many different enzymes, synthesis of these proteins may be reduced by lysine deficiency, thereby decreasing the formation of alanine, glutamate and glycine in the body. This view supports the concept that both the availability of substrates and enzyme activity affect endogenous synthesis of NEAA in animals (Hou et al. 2016b). A balanced and adequate supply of AAs in a lysine-adequate diet may promote the synthesis of NEAA to optimize protein synthesis in skeletal muscle. Growing evidence shows that NEAA play important roles in maximizing feed efficiency and muscle growth in livestock species (including pigs) and poultry (Hou et al. 2016b; Rezaei et al. 2013; Wang et al. 2013, 2014, 2015; Wu et al. 2011a, b, 2013; Yi et al. 2015). Further increase in dietary lysine from the adequate to the excess level did not further increase plasma concentrations of these 3 NEAA (Table 4). Under lysine-excess conditions, although lysine was not a limiting AA, there might be a second limiting AA as noted previously that might limit the de novo synthesis of proteinogenic NEAA. These findings, however, are in disagreement with those of Roy et al. (2000), who did not observe any difference in plasma concentrations of the aforementioned 3 NEAA among three dietary lysine levels. Zeng et al. (2013) only observed a decrease in plasma glutamate concentration with a lysine deficient, but not a lysine excess, diet fed to growing pigs. Furthermore, the plasma concentrations of alanine and glycine were not affected by the level of dietary lysine in their study (Zeng et al. 2013). Reason for the discrepancies between the present study and those previous studies might be due to the differences in the age of the pigs used. Early-stage growing pigs (BW ranging from 20 to 40 kg) were used by Roy et al. (2000) and Zeng et al. (2013), whereas late-stage finishing pigs (BW ranging from 90 to 130 kg) were used in this study.

Although the plasma concentrations of 2 EAA (methionine and tryptophan) and 9 NEAA (aspartate, β-alanine, cysteine in total, glutamine, ornithine, proline, serine, tyrosine and taurine) did not change in response to different intakes of dietary lysine, the plasma concentrations of leucine and asparagine followed two different patterns of change. Pattern 4 was observed for leucine and pattern 5 was for asparagine (Table 4). Although both of these patterns had some similarity to pattern 1, why plasma concentrations of leucine and asparagine followed those two slightly different patterns is unknown. For plasma leucine concentration, there was no significant difference between the pigs fed Diets II and III, whereas for plasma asparagine concentration, there was a difference between the pigs fed Diets II and III.

Methionine and tryptophan are two EAA, but their plasma concentrations did not change as other EAA in pattern 2 as described previously. In the pigs fed Diet I, the relatively excess methionine might have been metabolized via transmethylation reactions, with its methyl group being transferred to pathways for choline and creatine synthesis in the body (Wu 2013), while the excessive amount of tryptophan might have been degraded via the kynurenine pathway to form kynurenine in the liver, intestine, and lymphoid organs (Wu 2013). Further studies, however, are required to test this hypothesis.

## Conclusions

Results from the present study indicate that dietary levels of lysine can affect the plasma concentrations of 13 AAs (7 EAA and 6 NEAA) in late-stage finishing pigs in five distinct patterns. Plasma concentration of 7 AAs (threonine, histidine, phenylalanine, isoleucine, valine, arginine and citrulline) decreased with the lysine-adequate diet but not further decreased with the lysine-excess diet, when compared to the lysine-deficient diet. Among these AAs, arginine was decreased in the greatest proportion. We suggest that the skeletal muscle growth of late-stage finishing pigs may be further increased with a lysine-excess diet if the plasma concentrations of theses 7 AAs, primarily arginine, can be increased through dietary supplementation or other practical nutritional management strategies. Our findings underscore the importance of balanced and adequate provision of AAs in diets for optimizing lean-tissue growth in finishing pigs.

## Abbreviations

AAs: amino acids
ADG: average daily gain
BW: body weight
EAA: essential amino acid(s), excluding lysine
EDTA: ethylenediaminetetraacetic acid
GLM: general linear model
HPLC: high-performance liquid chromatography
NEAA: nonessential amino acid(s)
NRC: National Research Council
PDIFF: *P* values for differences
SAS: statistical analysis system
T: T test
TAA: total amino acid(s)
